# Arabidopsis *OSMOTIN 34* Functions in the ABA Signaling Pathway and Is Regulated by Proteolysis

**DOI:** 10.3390/ijms22157915

**Published:** 2021-07-24

**Authors:** Eun-Joo Park, Tae-Houn Kim

**Affiliations:** 1 Department of Bio-Health Convergence, Duksung Women’s University, Seoul 01369, Korea; cokun2013@duksung.ac.kr; 2 Department of Biotechnology, Duksung Women’s University, Seoul 01369, Korea

**Keywords:** *OSMOTIN 34*, *OSMOTIN 34-like*, abscisic acid, F-Box gene

## Abstract

Plants have evolutionarily established resistance responses to a variety of abiotic stress conditions, in which ABA mediates the integrated regulation of these stress responses. Numerous proteins function at the transcription level or at the protein level when contributing to controls of the ABA signaling process. Although osmotin is identified as a salt-inducible protein, its function in the abiotic stress response is yet to be elucidated. To examine the role of Arabidopsis *OSMOTIN 34* (*OSM34*) in the ABA signaling pathway, a deletion mutant *osm34* generated by a CRISPR/Cas9 system and the double mutant *osm34 osml* (*osmotin 34-like*) were analyzed for various ABA responses. Both *osm34* and *osm34 osml* showed reduced levels of ABA responses in seeds and leaves. Moreover, proline level and expression of the proline biosynthesis gene *P5CS1* was significantly reduced in *osm34 osml.* Interestingly, OSM34 binds to SKP2A, an F-Box protein whose transcription is induced by ABA. The protein stability of OSM34 was determined to be under the control of the 26S proteasome. In conclusion, our data suggest that OSM34 functions as a positive regulator in the generation of ABA responses and is under post-translational control.

## 1. Introduction

Under unfavorable conditions, plants need to cope with environmental stresses and alter their growth pattern to enhance viability. One of the major stresses affecting plant growth is drought, and the drought stress response is related to the ABA signaling pathway [1,2]. In upstream segments of the ABA signaling pathway, the ABA receptors PYR/PYL/RCARs interact with PP2Cs in the presence of ABA. This interaction induces the release of SnRK2s from the tethering of PP2Cs, resulting in activation of SnRK2s [3,4,5,6,7,8]. The activated SnRK2s subsequently phosphorylates several downstream targets, such as ABFs for gene induction and SLAC1 for stomata closure [1,2,4,5,9,10]. In addition, reactive oxygen species (ROS) also play an essential role as a second messenger [11]. To prevent plant damage by excessive accumulation of ROS, the produced ROS is eliminated either by antioxidants such as ascorbic acid and proline or by enzymatic degradation via ascorbate peroxidases, catalases, and glutathione peroxidases [11,12,13,14,15,16,17,18,19].

Osmotic stress is tightly linked to various abiotic stress conditions such as drought, and is modulated by multiple pathways [1,2,20,21]. Osmotin was first discovered to be induced in salt-adapted tobacco cells, and in response to ABA and drought [22,23,24]. Interestingly, the transcripts level of *osmotin* increases after exposure to ABA, but the protein level remains unchanged, suggesting the presence of a post-translational regulatory mechanism of *osmotin* [24,25]. *Osmotin* belongs to the *PR5* (*Pathogenesis Related 5*) family, and several studies have reported that osmotin is involved in the generation of biotic stress responses [26,27,28,29,30]. In Arabidopsis, the *osmotin-like* gene *OSMOTIN 34* (*OSM34*) was first identified in the screening of the Arabidopsis cDNA library using tobacco *osmotin* as a probe [31]. However, its function under the abiotic stress signaling pathway is yet to be elucidated. Given that transgenic lines constitutively expressing the *osmotin* gene (and its homologs) in various plants have enhanced tolerance to abiotic stress, the putative function of *OSM34* was predicted to be related to the ABA signaling pathway [32,33,34,35,36]. However, genetic evidence using knockout or knockdown mutants to characterize the function of *OSM34* remains unaccomplished owing to the lack of available T-DNA mutants.

Recent studies have shown that specific degradation of regulatory proteins plays an important role in the control of ABA signaling pathways. Among the components involved in the ubiquitin-mediated pathways, the diverse E3 ligase reportedly participates in specific target protein recognition [37]. For instance, ABA receptors PYR1 and PYL4 are targeted for degradation by RSL1. A mutation in *RSL1* enhances sensitivity to ABA, thereby implying a negative role in the ABA signaling pathway [38]. Moreover, the transcription factor ABI5 is controlled by various E3 ligase such as KEG, RPN10, DWA1/2, and ABD1 [39,40,41,42,43]. These results indicate that the ABA signaling pathway is largely controlled by ubiquitin-mediated proteolysis. Although more than 700 F-box genes exist in Arabidopsis, studies of the F-box gene that function in the ABA signaling pathway have been limited.

In this report, we present phenotypes of defective ABA responses produced by mutations introduced in the Arabidopsis gene *OSMOTIN 34* and its homologous gene *OSMOTIN 34-LIKE*. Moreover, we identified that F-box SKP2A, which is transcriptionally induced by ABA, interacts with OSM34, indicating post-translational regulation of OSM34.

## 2. Results

### 2.1. The osm34 Mutant Is a Deletion Mutant of OSM34 Generated by a CRISPR/Cas9 Approach

**Figure 1:**
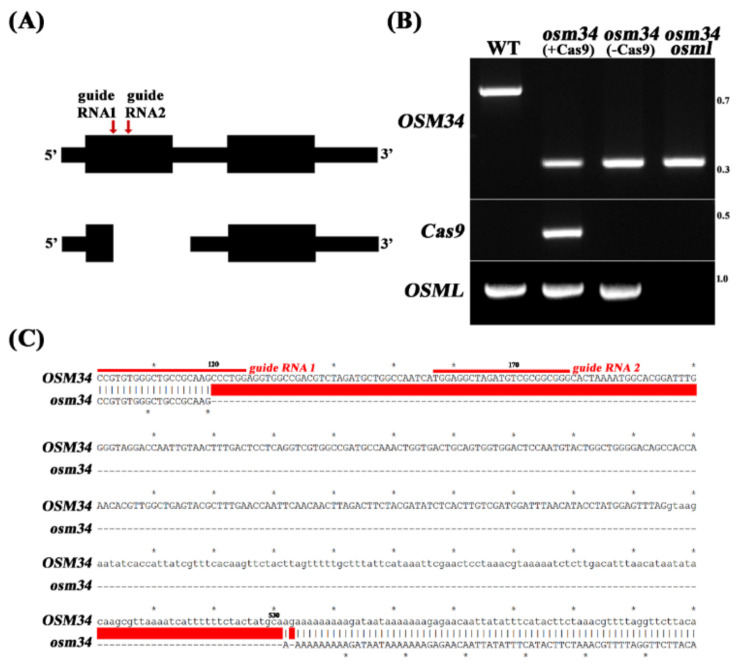
Generation of the deletion mutant *osm34* by a CRISPR/Cas9 approach (**A**) Two guide RNAs targeting first exon region of *OSM34* were designed (red line with arrow heads, top), and a deletion mutant *osm34* was isolated (bottom). (**B**) PCR genotyping confirmed a large deletion in *osm34* and the removal of Cas9 by crossing into wild-type (Col-0). By crossing Cas9-free *osm34* into *osml* (*osmotin 34-like*), the double mutant *osm34 osml* was generated. (**C**) Sequence analyses of *osm34* revealed location of the deletion when compared to the wild-type sequences. The red color bar indicates the deleted sequences in *osm34*.

In order to elucidate the function of *osmotin* genes in the regulation of ABA signal transduction, we investigated two genetic mutants of Arabidopsis: *OSMOTIN 34* (*OSM34*) (AT4G11650) and *OSMOTIN 34-LIKE* (*OSML*) (AT1G77700). *OSML* (AT1G77700) was selected based on the amino acid sequence similarities (Supplementary Figure S1). Since no knockout mutant of *OSM34* was available from public sites, we applied the CRISPR/Cas9 approach to generate a deletion mutant, *osm34*. By introducing two guide RNAs targeting two independent sites in the first exon of *OSM34* (Figure 1A), a deletion mutant of *OSM34* (*osm34*) was isolated and found to have a 413 bp deletion (Figure 1B,C). *osm34* revealed PCR bands with a shorter length in different tissues and in subsequent generations (data not shown). To minimize the putative off-target effect by Cas9 [44], a Cas9-free *osm34* was generated by crossing *osm34* into the wild-type (Col-0) control (Figure 1B).

### 2.2. The osm34 Mutant Produced Defects in the Generation of ABA Responses

**Figure 2:**
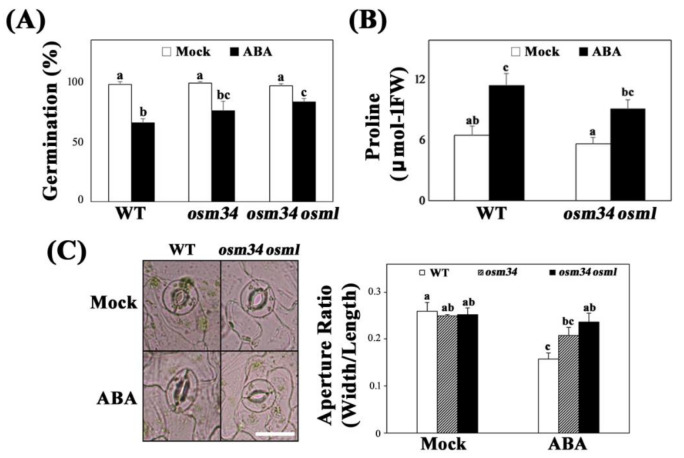
The mutant *osm34* and *osm34 osml* produce defects in the generation of ABA responses (**A**) Seed germination assay using *osm34* and *osm34 osml* under 0.5 μM ABA. a/b/bc/c indicate statistically different groups based on one-way ANOVA Duncan post-hoc test (*p* < 0.05). (**B**) Quantification of proline level using 1.25% ninhydrin solution. Three-week-old rosette leaves of wild-type (Col-0) and *osm34 osml* were sprayed with 30 μM ABA for two days. a/ab/bc/c indicate different groups based on one-way ANOVA Duncan post-hoc test (*p* < 0.05). (**C**) The ABA-induced stomatal closure was analyzed using four-week-old wild-type (Col-0), *osm34*, and *osm34 osml* after 2 h of exposure to 10 μM ABA. a/ab/bc/c groups based on one-way ANOVA Duncan post-hoc test (*p* < 0.05).

Following isolation of the Cas9-free *osm34*, we subsequently generated a double mutant *osm34 osml* (*osmotin 34-like*) to minimize the problem of genetic redundancy of *OSM34-like* genes (Figure 1B). *osm34* showed reduced responses to ABA in seed germination and stomatal closing assays (Figure 2A,C). However, root growth inhibition caused by ABA, salt, and mannitol was not affected by the mutation in *OSM34* (Supplementary Figure S2). Reduced ABA responses in seeds and leaves were greater in *osm34 osml* than in the single mutant *osm34* or the wild-type control (Figure 2A,C).

**Figure 3:**
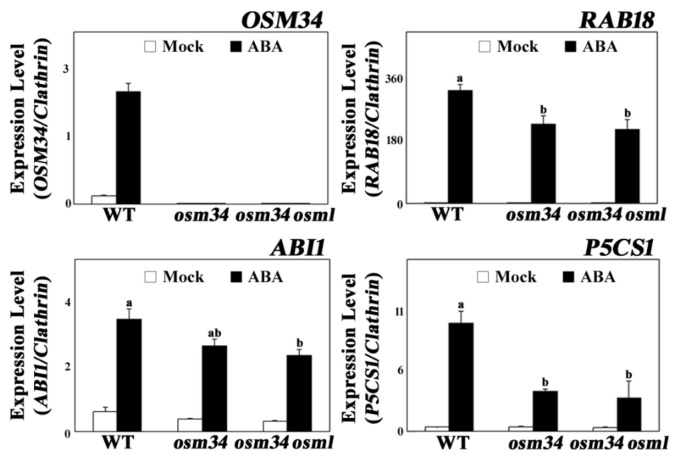
The mutants *osm34* and *osm34 osml* exhibit reduced ABA induction of gene expression Six-day-old seedlings were treated with 50 μM ABA for 24 h and used for quantitative PCR analyses. Clathrin was used as an internal control. a/b/ab indicate different groups according to one-way ANOVA Duncan post-hoc test (*p* < 0.05).

Similar to the physiological changes in *osm34* and *osm34 osml*, the ABA induction of ABA-responsive genes, including *RAB18*, *ABI1*, and *P5CS1*, were strongly inhibited by defects in the *OSM34* and *OSML* genes (Figure 3). The lack of *OSM34* transcripts in *osm34* or *osm34 osml* with one of the primers binding in the deleted region and the presence of a putative stop codon (at the 152nd amino acid position) generated by the deletion in *osm34* indicate that *osm34* is a true homozygous mutant producing no functional OSM34. Moreover, transgenic lines with reduced expression of *OSM34* by an artificial *miRNA* approach produced a similar pattern of inhibition for ABA-responsive gene expression (Supplementary Figure S3). Consistent with the reduced ABA induction of *P5CS1* involved in proline biosynthesis, the endogenously induced proline level by ABA was significantly lower in *osm34 osml* compared to the wild-type control (Figure 2B). This indicates that the function of *OSM34* in the generation of ABA responses is correlated with the ABA-induced proline accumulation. Taken together, our results suggest that *OSM34* functions positively in the ABA signaling pathway during seed germination and stomatal movement via control of gene expression and proline levels.

### 2.3. The Protein Stability of OSM34 Is Regulated by SKP2A in a 26S Proteasome-Mediated Pathway

**Figure 4:**
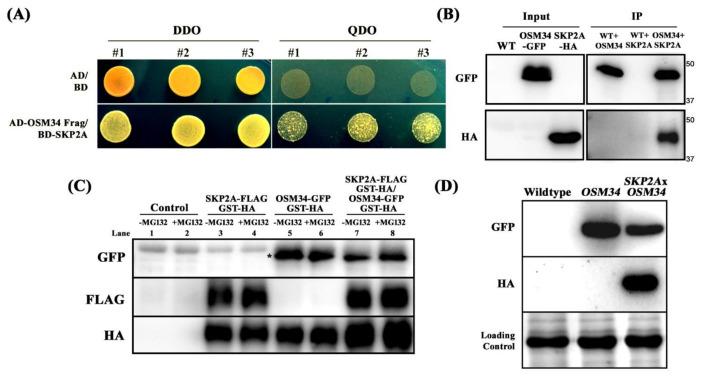
OSM34 interacts with SKP2A and is destabilized via 26S proteasome-dependent pathway (**A**) Yeast two-hybrid assay showed direct interaction between OSM34 (42-244) and SKP2A (1-360). Co-transformed yeast colonies were grown on either DDO media (-Leu/-Trp) or QDO media (-Ade/-His/-Leu/-Trp) containing 10 mM 3-AT. (**B**) The Arabidopsis transgenic lines expressing either *OSM34-GFP* or *SKP2A-HA* were used for co-immunoprecipitation (co-IP) analysis. Input was collected before co-IP analysis. After immunoprecipitation with anti-GFP-sepharose beads, Western blot analysis was performed on the co-IP sample using anti-HA antibody. (**C**) Cell-free OSM34 degradation assay using tobacco leaves with transiently expressing either SKP2A-FLAG/GST-HA or OSM34-GFP/GST-HA. GST-HA was co-expressed as an internal control for each transient expression. Total proteins were incubated for 3 h with 20 μM MG132 or DMSO and subsequently analyzed by Western blot. * indicates the target GFP band. (**D**) In vivo stability of OSM34 was analyzed by Western blot assays using the transgenic lines co-expressing *OSM34-GFP* and *SKP2A-HA* or expressing *OSM34-GFP* alone.

Applying yeast two-hybrid assays, N-terminal-deleted OSM34 (42-244) was identified to interact with SKP2A (1-360) (Figure 4A). The specific interaction between OSM34 and SKP2A was further analyzed using co-immunoprecipitation (co-IP) assays (Figure 4B). After mixing two protein extracts from the transgenic lines expressing either *OSM34-GFP* or *SKP2A-HA*, pull down of SKP2A as an interactive partner of OSM34 was achieved by co-IP of OSM34 with a anti-GFP antibody (Figure 4B). Since SKP2A as a component of SCF E3 ligase was shown to interact with E2FC and subsequently degrade it [45], it was deliberated whether the interaction of SKP2A with OSM34 leads to the specific protein degradation of OSM34. To examine this hypothesis, total protein was extracted from tobacco leaves transiently expressing either *OSM34-GFP*/*GST-HA* or *SKP2A-FLAG*/*GST-HA*, and was applied for cell-free degradation assays. Incubation with SKP2A (lane 7) showed significantly reduced protein level of OSM34, as compared to the OSM34 alone (lane 5) (Figure 4C). To investigate whether the reduced protein level of OSM34 after co-incubation with SKP2A is mediated by the 26S proteasome-dependent pathway, we examined the protein stability of OSM34 after exposure to MG132 (Figure 4C). Increased level of OSM34 with MG132 treatment (lane 8) was observed, compared to the control without MG132 (lane 7) (Figure 4C). Furthermore, in vivo protein level of OSM34 was highly reduced in the transgenic line co-expressing OSM34 and SKP2A, as compared to the line expressing OSM34 alone (Figure 4D). Results of the *in vitro* and in vivo assays to determine the stability of OSM34 indicate that the specific interaction of OSM34 with SKP2A induces the proteolysis of OSM34 in a 26S proteasome-dependent pathway.

## 3. Discussion

Although major components of the ABA signaling pathway have been identified, many regulatory proteins linking ABA signaling to environmental stress-resistant responses have yet to be identified. One such regulatory protein, osmotin, was previously reported to be induced under salt and ABA stress at the transcriptional level, and was suggested to be controlled by an unknown post-transcriptional regulation [25]. In order to determine whether osmotin functions in ABA signal transduction, and how osmotin is regulated at the protein level, genetic mutations were introduced on the *osmotin* genes and investigated. The mutant *osm34* was generated by a CRISPR/Cas9 approach, and the double mutant *osm34 osml* was constructed by crossing *osm34* into a T-DNA mutant of *OSMOTIN 34-LIKE* (*OSML*) (Figure 1). The mutant *osm34* produced phenotypes with reduced ABA responses in seed germination, stomatal movement, and gene expression, whereas the reduced ABA responses in *osm34 osml* were more pronounced as compared to *osm34* (Figure 2 and Figure 3). This implies that *OSM34* and *OSML* function at least additively as positive regulators in certain pathways of ABA signal transduction. The introduction of additional mutations to other *OSM34-LIKE* genes will contribute to understanding the detailed function and interaction of each *osmotin* gene. Because *RAB18* and *ABI1* have been known as ABA-responsive genes [46,47], reduced ABA induction of both genes in *osm34* and *osm34 osml* indicates that *OSM34* may function in the positive regulation of ABA-induced gene expression. Downregulation of *OSM34* in the transgenic lines generated by an artificial *miRNA* approach also induced similar gene expression results as *osm34*, especially for *P5CS1* (Supplementary Figure S3). Taken together, these results indicate that a mutation in *OSM34* indeed causes defects in ABA signal transduction. One of the possible functions of *OSM34* in the ABA signaling pathway is to regulate proline levels, considering that the *P5CS1* expression was decreased in *osm34 osml* in conjunction with ABA (Figure 3), and the endogenous proline level of the ABA-treated *osm34 osml* was lower than the control (Figure 2B). This result coincides with previous reports which state that constitutive expression of osmotin in tobacco and soybean leads to enhanced proline accumulation [33,48]. Considering that increased prolines under abiotic stress could function as ROS scavengers, the reduced expression of *P5CS1* in *osm34 osml* implies that the control of proline levels and oxidative stress responses are directly affected by *OSM34*. However, confirming that conclusion would require the tissue-specific expression analysis of *P5CS1* and the measurement of proline levels at various time points.

To investigate a post-translational regulation of *osmotin*, we examined the protein stability of OSM34 and a possible E3 ligase for OSM34. The F-Box gene is known to play an important role in the signaling of numerous plant hormones [49], but its function in ABA signaling is largely unknown. By searching *F-box* genes that are transcriptionally induced by ABA treatment, we found *SKP2A* (AT1G21410) and *SKP2B* (AT1G77000) as candidate genes that function during ABA signal transduction (Supplementary Figure S4). Previously, *SKP2A* was discovered as a cell cycle regulator by targeting the repressor E2FC for the 26S proteasome-mediated degradation [45,50,51]. Considering that multiple targets of F-box have been reported in yeasts [52,53], *SKP2A* probably has additional targets that are involved in abiotic stress responses. Interestingly, SKP2A binds to OSM34 in yeast two-hybrid assays (Figure 4A). The physical interaction of OSM34 with SKP2A in yeasts was further confirmed in plants by co-immunoprecipitation assays (Figure 4B). Considering that SKP2A is a component of the SCF E3 ligase, the interaction of OSM34 with SKP2A could lead to specific degradation of OSM34. Degradation of the transiently expressed OSM34 was promoted by co-expression of SKP2A, and this SKP2A-dependent degradation of OSM34 was blocked by MG132 treatment in tobacco leaves (Figure 4C). Similar to the cell-free degradation assays, the OSM34 level in the transgenic line was reduced when crossed with the *SKP2A* overexpression line (Figure 4D), thereby supporting the premise that SKP2A mediates OSM34 degradation in vivo. These results indicate that OSM34 is post-translationally controlled by SKP2A in a 26S proteasome-dependent pathway.

In conclusion, our data suggest that *OSM34* functions in the generation of ABA responses, including the regulation of ABA–induced proline synthesis. Moreover, the protein stability of OSM34 is controlled by SKP2A in a 26S proteasome-dependent way.

## 4. Materials and Methods

### 4.1. Plants Materials

Surface-sterilized seeds of *Arabidopsis thaliana* wild-type (Col-0) and *osml* (AT1G77700, SALK_013068C) were sowed on growth media containing half-strength Murashige and Skoog salt (Sigma) with 1% sucrose (pH 5.8) and stratified at 4 °C for two days. Plants were grown under long-day condition (23 °C 16 h light/21 °C 8 h dark).

### 4.2. Construction of CRISPR/Cas9-Mediated Mutant and Transgenic Lines

To generate a deletion-prone mutation in the *OSM34* gene, two guide RNAs (each targeting different sites of *OSM34*) were designed (https://crispr.dbcls.jp, (16 March 2016)) [54] and merged into one plasmid containing Cas9. By analyzing more than 50 independent lines, a deletion mutant *osm34* was selected and analyzed by sequencing the *OSM34* gene. To minimize an off-target effect caused by Cas9, *osm34* was backcrossed into the wild-type (Col-0) two times, with subsequent isolation of the Cas9-free *osm34*. The *OSM34* knockdown lines were generated by an artificial *microRNA (amiRNA)* approach, wherein a PCR-tailored *amiRNA* construct targeting *OSM34* was generated based on pRS300 and transferred into the binary vector [55]. The Arabidopsis transgenic lines constitutively expressing either *OSM34-GFP* or *SKP2A-HA* under the control of the 35S promoter were also generated. For protein stability assays, the *OSM34* overexpression line was crossed into wild-type (Col-0) or the *SKP2A* overexpression line.

### 4.3. Germination/Root Growth Assay

Surface sterilized seeds were sown on growth media containing ABA (Sigma-Aldrich, CA) or ethanol (Merck, DE). Seed germination was examined at the indicated time after two days of stratification at 4 °C. For root growth assay, seedlings were vertically grown for five days, followed by transfer to control or ABA media. Elongated root length after the transfer was measured using Image J (NIH). Three biological replicates were analyzed.

### 4.4. Assay of Stomatal Closure

Four-week-old samples were covered with a dome for two days before the assay. Rosette leaves were excised and dipped into the opening buffer (10 mM MES, 5 mM KCl, 50 μM CaCl_2_, pH 6.15) for two hours, followed by incubation with ABA solution for another 2 h. Images of stomata were taken using an optical microscope mounted with a camera. The widths and lengths of stomata apertures were measured using Image J (NIH), and the ratio was calculated. Three biological replicates were performed and repeated three times (*n* = 3 × 3).

### 4.5. RNA Isolation and Gene Expression Analysis

Total RNA was isolated from six-day-old seedlings using the TRI Reagent^®^ (MRC, US). Recombinant DNase I (Takara, CN) was added, as per the manufacturer’s instructions. The isolated RNA (1 μg) was used for cDNA synthesis (Bioline, GB), and the synthesized cDNA was subjected to quantitative real-time PCR using SensiFAST™ SYBR^®^ Hi-ROX Kit (Bioline, GB). The relative expression of each gene was calculated based on the *Clathrin* level. The primer sequences used in this experiment were listed in Supplementary Table S1. 

### 4.6. Proline Quantification

Proline level was quantified applying the 1.25% ninhydrin solution method, with slight modification [56,57]. Briefly, three-week-old rosette leaves of wild-type (Col-0) and *osm34 osml* were sprayed with 30 μM ABA, and aerial parts were excised on the third day and used for ethanol extraction. The ethanol extracts were then mixed with 1.25% ninhydrin in glacial acetic acid and boiled for 30 min. Subsequently, absorbance was measured at 508 nm. Proline (Sigma, JP) concentration of each sample was calculated based on the standard curve. Three biological replicates were measured for each experiment and repeated three times (*n* = 3 × 3).

### 4.7. Yeast Two-Hybrid Assay

For the yeast two-hybrid assay, a part of cDNA encoding for the N-terminal-deleted OSM34 (42-244) and a full-length cDNA encoding for SKP2A (1-360) were cloned into the pGADT7 and pGBKT7 vectors, respectively. Growth conditions and media selection were followed according to the manufacturer’s instruction (Clontech). Each recombinant plasmid and control vector was co-transformed into AH109 using the electroporation method [58]. Double dropout media (-Leu/-Trp) was used to select colonies containing both pGADT7/pGBKT7 and pGADT7-OSM34/pGBKT7-SKP2A. Colonies with positive interaction were selected by the quadruple dropout media (-Ade/-His/-Leu/-Trp).

### 4.8. Co-Immunoprecipitation and Western Blot Analysis

Seven-day-old seedlings of transgenic lines constitutively expressing either *SKP2A-HA* or *OSM34-GFP* were subjected to total protein extraction using the protein extraction buffer (150 mM NaCl, 50 mM Tris pH 7.5, 1 × protease inhibitor (Roche, DE), 1 mM PMSF, 0.01% NP-40, 10 mM MgCl_2_, 10% glycerol). Co-immunoprecipitation was performed at 4 °C overnight, using anti-GFP-Sepharose beads (Abcam, GB). Beads were subsequently washed four times, and the immunoprecipitated proteins were subjected to Western blot analysis with primary antibodies (GFP (B-2) Santa Cruz Biotech, US; HA-HRP (3F10) Roche, CH; FLAG Cell Signaling Technology, US), followed by incubation with secondary antibody (anti-mouse HRP conjugated Pierce^®^, US). The blot was then developed using Clarity^TM^ Western ECL solution (Bio-Rad, US).

### 4.9. In Vitro and In Vivo Protein Degradation Assays

Transient expression of each construct in tobacco for cell-free degradation analysis was performed using the Agrobacterium-mediated transient gene expression method, with slight modification [59]. Briefly, two days after infiltration, total proteins were extracted using the protein extraction buffer. Total protein extracts were mixed as designated, and incubated for 3 h at room temperature. The reaction was terminated by adding 5× sample buffer, followed by boiling the mixture. For the in vivo OSM34 degradation assay, total proteins extracted from wild-type (Col-0) and transgenic lines expressing *OSM34-GFP* alone or co-expressing *OSM34-GFP* and *SKP2A-HA* were incubated for 3 h, and subsequently subjected to Western blot analysis.

## Supplementary Materials

The following are available online at https://www.mdpi.com/article/10.3390/ijms22157915/s1, Figure S1. Phylogenetic tree of OSM34 and similar proteins. Figure S2: Root growth inhibition caused by abiotic stresses was not affected by the mutation in *OSM34*. Figure S3: The *OSM34* knockdown lines generated by an *amiRNA* approach show reduced ABA induction of gene expression. Figure S4: *F-Box* genes *SKP2A* and *SKP2B* are transcriptionally induced by ABA treatment. Table S1: List of primers used in this work.

## References

[1] Cutler S.R., Rodriguez P.L., Finkelstein R.R., Abrams S.R. (2010). Abscisic acid: Emergence of a core signaling network. Annu. Rev. Plant. Biol..

[2] Kim T.H., Bohmer M., Hu H., Nishimura N., Schroeder J.I. (2010). Guard cell signal transduction network: Advances in understanding abscisic acid, CO2, and Ca2+ signaling. Annu. Rev. Plant. Biol..

[3] Fujii H., Chinnusamy V., Rodrigues A., Rubio S., Antoni R., Park S.Y., Cutler S.R., Sheen J., Rodriguez P.L., Zhu J.K. (2009). In vitro reconstitution of an abscisic acid signalling pathway. Nature.

[4] Park S.Y., Fung P., Nishimura N., Jensen D.R., Fujii H., Zhao Y., Lumba S., Santiago J., Rodrigues A., Chow T.F. (2009). Abscisic acid inhibits type 2C protein phosphatases via the PYR/PYL family of START proteins. Science.

[5] Ma Y., Szostkiewicz I., Korte A., Moes D., Yang Y., Christmann A., Grill E. (2009). Regulators of PP2C phosphatase activity function as abscisic acid sensors. Science.

[6] Nishimura N., Hitomi K., Arvai A.S., Rambo R.P., Hitomi C., Cutler S.R., Schroeder J.I., Getzoff E.D. (2009). Structural mechanism of abscisic acid binding and signaling by dimeric PYR1. Science.

[7] Nishimura N., Sarkeshik A., Nito K., Park S.Y., Wang A., Carvalho P.C., Lee S., Caddell D.F., Cutler S.R., Chory J. (2010). PYR/PYL/RCAR family members are major in-vivo ABI1 protein phosphatase 2C-interacting proteins in Arabidopsis. Plant. J..

[8] Santiago J., Dupeux F., Round A., Antoni R., Park S.Y., Jamin M., Cutler S.R., Rodriguez P.L., Marquez J.A. (2009). The abscisic acid receptor PYR1 in complex with abscisic acid. Nature.

[9] Antoni R., Gonzalez-Guzman M., Rodriguez L., Rodrigues A., Pizzio G.A., Rodriguez P.L. (2012). Selective inhibition of clade A phosphatases type 2C by PYR/PYL/RCAR abscisic acid receptors. Plant. Physiol..

[10] Brandt B., Brodsky D.E., Xue S., Negi J., Iba K., Kangasjarvi J., Ghassemian M., Stephan A.B., Hu H., Schroeder J.I. (2012). Reconstitution of abscisic acid activation of SLAC1 anion channel by CPK6 and OST1 kinases and branched ABI1 PP2C phosphatase action. Proc. Natl. Acad. Sci. USA.

[11] Waszczak C., Carmody M., Kangasjarvi J. (2018). Reactive oxygen species in plant signaling. Annu. Rev. Plant. Biol..

[12] Asada K. (2006). Production and scavenging of reactive oxygen species in chloroplasts and their functions. Plant. Physiol..

[13] Roxas V.P., Smith R.K., Allen E.R., Allen R.D. (1997). Overexpression of glutathione S-transferase/glutathione peroxidase enhances the growth of transgenic tobacco seedlings during stress. Nat. Biotechnol..

[14] Willekens H., Chamnongpol S., Davey M., Schraudner M., Langebartels C., Van Montagu M., Inze D., Van Camp W. (1997). Catalase is a sink for H2O2 and is indispensable for stress defence in C3 plants. EMBO J..

[15] Noctor G., Foyer C.H. (1998). Ascorbate and Glutathione: Keeping active oxygen under control. Annu. Rev. Plant. Physiol. Plant. Mol. Biol..

[16] Hayat S., Hayat Q., Alyemeni M.N., Wani A.S., Pichtel J., Ahmad A. (2012). Role of proline under changing environments: A review. Plant. Signal. Behav..

[17] Golldack D., Li C., Mohan H., Probst N. (2014). Tolerance to drought and salt stress in plants: Unraveling the signaling networks. Front. Plant. Sci.

[18] Asad M.A.U., Zakari S.A., Zhao Q., Zhou L., Ye Y., Cheng F. (2019). Abiotic stresses intervene with ABA signaling to induce destructive metabolic pathways leading to death: Premature leaf senescence in plants. Int. J. Mol. Sci..

[19] Sandalio L.M., Romero-Puertas M.C. (2015). Peroxisomes sense and respond to environmental cues by regulating ROS and RNS signalling networks. Ann. Bot..

[20] Bray E.A. (1993). Molecular responses to water deficit. Plant. Physiol..

[21] Anil Kumar S., Hima Kumari P., Shravan Kumar G., Mohanalatha C., Kavi Kishor P.B. (2015). Osmotin: A plant sentinel and a possible agonist of mammalian adiponectin. Front. Plant. Sci..

[22] Larosa P.C., Singh N.K., Hasegawa P.M., Bressan R.A. (1989). Stable NaCl tolerance of tobacco cells is associated with enhanced accumulation of osmotin. Plant. Physiol..

[23] Singh N.K., Bracker C.A., Hasegawa P.M., Handa A.K., Buckel S., Hermodson M.A., Pfankoch E., Regnier F.E., Bressan R.A. (1987). Characterization of osmotin: A thaumatin-like protein associated with osmotic adaptation in plant cells. Plant. Physiol..

[24] Singh N.K., Nelson D.E., Kuhn D., Hasegawa P.M., Bressan R.A. (1989). Molecular cloning of osmotin and regulation of its expression by ABA and adaptation to low water potential. Plant. Physiol..

[25] Larosa P.C., Chen Z., Nelson D.E., Singh N.K., Hasegawa P.M., Bressan R.A. (1992). Osmotin gene expression is posttranscriptionally regulated. Plant. Physiol..

[26] Ibeas J.I., Yun D.J., Damsz B., Narasimhan M.L., Uesono Y., Ribas J.C., Lee H., Hasegawa P.M., Bressan R.A., Pardo J.M. (2001). Resistance to the plant PR-5 protein osmotin in the model fungus Saccharomyces cerevisiae is mediated by the regulatory effects of SSD1 on cell wall composition. Plant. J..

[27] Yun D.J., Ibeas J.I., Lee H., Coca M.A., Narasimhan M.L., Uesono Y., Hasegawa P.M., Pardo J.M., Bressan R.A. (1998). Osmotin, a plant antifungal protein, subverts signal transduction to enhance fungal cell susceptibility. Mol. Cell.

[28] Liu D., Raghothama K.G., Hasegawa P.M., Bressan R.A. (1994). Osmotin overexpression in potato delays development of disease symptoms. Proc. Natl. Acad. Sci. USA.

[29] Monteiro S., Barakat M., Picarra-Pereira M.A., Teixeira A.R., Ferreira R.B. (2003). Osmotin and thaumatin from grape: A putative general defense mechanism against pathogenic fungi. Phytopathology.

[30] Salzman R.A., Koiwa H., Ibeas J.I., Pardo J.M., Hasegawa P.M., Bressan R.A. (2004). Inorganic cations mediate plant PR5 protein antifungal activity through fungal Mnn1- and Mnn4-regulated cell surface glycans. Mol. Plant. Microbe Interact..

[31] Capelli N., Diogon T., Greppin H., Simon P. (1997). Isolation and characterization of a cDNA clone encoding an osmotin-like protein from Arabidopsis thaliana. Gene.

[32] Bhattacharya A., Saini U., Joshi R., Kaur D., Pal A.K., Kumar N., Gulati A., Mohanpuria P., Yadav S.K., Kumar S. (2014). Osmotin-expressing transgenic tea plants have improved stress tolerance and are of higher quality. Transgenic Res..

[33] Subramanyam K., Arun M., Mariashibu T.S., Theboral J., Rajesh M., Singh N.K., Manickavasagam M., Ganapathi A. (2012). Overexpression of tobacco osmotin (Tbosm) in soybean conferred resistance to salinity stress and fungal infections. Planta.

[34] Viktorova J., Rehorova K., Musilova L., Suman J., Lovecka P., Macek T. (2017). New findings in potential applications of tobacco osmotin. Protein Expr. Purif..

[35] Weber R.L., Wiebke-Strohm B., Bredemeier C., Margis-Pinheiro M., de Brito G.G., Rechenmacher C., Bertagnolli P.F., de Sa M.E., Campos Mde A., de Amorim R.M. (2014). Expression of an osmotin-like protein from Solanum nigrum confers drought tolerance in transgenic soybean. BMC Plant. Biol..

[36] Zhang Y., Shih D.S. (2007). Isolation of an osmotin-like protein gene from strawberry and analysis of the response of this gene to abiotic stresses. J. Plant. Physiol..

[37] Craig K.L., Tyers M. (1999). The F-box: A new motif for ubiquitin dependent proteolysis in cell cycle regulation and signal transduction. Prog. Biophys. Mol. Biol..

[38] Bueso E., Rodriguez L., Lorenzo-Orts L., Gonzalez-Guzman M., Sayas E., Munoz-Bertomeu J., Ibanez C., Serrano R., Rodriguez P.L. (2014). The single-subunit RING-type E3 ubiquitin ligase RSL1 targets PYL4 and PYR1 ABA receptors in plasma membrane to modulate abscisic acid signaling. Plant. J..

[39] Lee J.H., Yoon H.J., Terzaghi W., Martinez C., Dai M., Li J., Byun M.O., Deng X.W. (2010). DWA1 and DWA2, two Arabidopsis DWD protein components of CUL4-based E3 ligases, act together as negative regulators in ABA signal transduction. Plant. Cell.

[40] Seo K.I., Lee J.H., Nezames C.D., Zhong S., Song E., Byun M.O., Deng X.W. (2014). ABD1 is an Arabidopsis DCAF substrate receptor for CUL4-DDB1-based E3 ligases that acts as a negative regulator of abscisic acid signaling. Plant. Cell.

[41] Smalle J., Kurepa J., Yang P., Emborg T.J., Babiychuk E., Kushnir S., Vierstra R.D. (2003). The pleiotropic role of the 26S proteasome subunit RPN10 in Arabidopsis growth and development supports a substrate-specific function in abscisic acid signaling. Plant. Cell.

[42] Liu H., Stone S.L. (2010). Abscisic acid increases Arabidopsis ABI5 transcription factor levels by promoting KEG E3 ligase self-ubiquitination and proteasomal degradation. Plant. Cell.

[43] Stone S.L., Williams L.A., Farmer L.M., Vierstra R.D., Callis J. (2006). Keep on going, a RING E3 ligase essential for Arabidopsis growth and development, is involved in abscisic acid signaling. Plant. Cell.

[44] Fu Y., Foden J.A., Khayter C., Maeder M.L., Reyon D., Joung J.K., Sander J.D. (2013). High-frequency off-target mutagenesis induced by CRISPR-Cas nucleases in human cells. Nat. Biotechnol..

[45] Del Pozo J.C., Boniotti M.B., Gutierrez C. (2002). Arabidopsis E2Fc functions in cell division and is degraded by the ubiquitin-SCF(AtSKP2) pathway in response to light. Plant. Cell.

[46] Jeannette E., Rona J.P., Bardat F., Cornel D., Sotta B., Miginiac E. (1999). Induction of RAB18 gene expression and activation of K+ outward rectifying channels depend on an extracellular perception of ABA in Arabidopsis thaliana suspension cells. Plant. J..

[47] Leung J., Merlot S., Giraudat J. (1997). The Arabidopsis abscisic acid-insensitive2 (ABI2) and ABI1 genes encode homologous protein phosphatases 2C involved in abscisic acid signal transduction. Plant. Cell.

[48] Barthakur S., Babu V., Bansa K.C. (2001). Over-expression of Osmotin induces proline accumulation and confers tolerance to osmotic stress in transgenic tobacco. J. Plant Biochem. Biotechnol..

[49] Yu H., Wu J., Xu N., Peng M. (2007). Roles of F-box proteins in plant hormone responses. Acta Biochim. Biophys. Sin..

[50] Del Pozo J.C., Diaz-Trivino S., Cisneros N., Gutierrez C. (2006). The balance between cell division and endoreplication depends on E2FC-DPB, transcription factors regulated by the ubiquitin-SCFSKP2A pathway in Arabidopsis. Plant. Cell.

[51] Jurado S., Diaz-Trivino S., Abraham Z., Manzano C., Gutierrez C., del Pozo C. (2008). SKP2A, an F-box protein that regulates cell division, is degraded via the ubiquitin pathway. Plant J..

[52] Bai C., Sen P., Hofmann K., Ma L., Goebl M., Harper J.W., Elledge S.J. (1996). SKP1 connects cell cycle regulators to the ubiquitin proteolysis machinery through a novel motif, the F-box. Cell.

[53] Meimoun A., Holtzman T., Weissman Z., McBride H.J., Stillman D.J., Fink G.R., Kornitzer D. (2000). Degradation of the transcription factor Gcn4 requires the kinase Pho85 and the SCF(CDC4) ubiquitin-ligase complex. Mol. Biol. Cell.

[54] Naito Y., Hino K., Bono H., Ui-Tei K. (2015). CRISPRdirect: Software for designing CRISPR/Cas guide RNA with reduced off-target sites. Bioinformatics.

[55] Schwab R., Ossowski S., Riester M., Warthmann N., Weigel D. (2006). Highly specific gene silencing by artificial microRNAs in Arabidopsis. Plant. Cell.

[56] Shabnam N., Tripathi I., Sharmila P., Pardha-Saradhi P. (2016). A rapid, ideal, and eco-friendlier protocol for quantifying proline. Protoplasma.

[57] Cross J.M., von Korff M., Altmann T., Bartzetko L., Sulpice R., Gibon Y., Palacios N., Stitt M. (2006). Variation of enzyme activities and metabolite levels in 24 Arabidopsis accessions growing in carbon-limited conditions. Plant Physiol..

[58] Thompson J.R., Register E., Curotto J., Kurtz M., Kelly R. (1998). An improved protocol for the preparation of yeast cells for transformation by electroporation. Yeast.

[59] Shamloul M., Trusa J., Mett V., Yusibov V. (2014). Optimization and utilization of Agrobacterium-mediated transient protein production in Nicotiana. J. Vis. Exp..

