# Supplementary material for: Arabidopsis OSMOTIN 34 Functions in the ABA Signaling Pathway and Is Regulated by Proteolysis
Source: Int J Mol Sci. 2021 Jul 24;22(15):7915. doi: 10.3390/ijms22157915 (PMC8347876; doi:10.3390/ijms22157915)
Supplement: Supplementary file 1 [file ijms-22-07915-s001.zip › Supplementary Figures S1-S4.pdf]

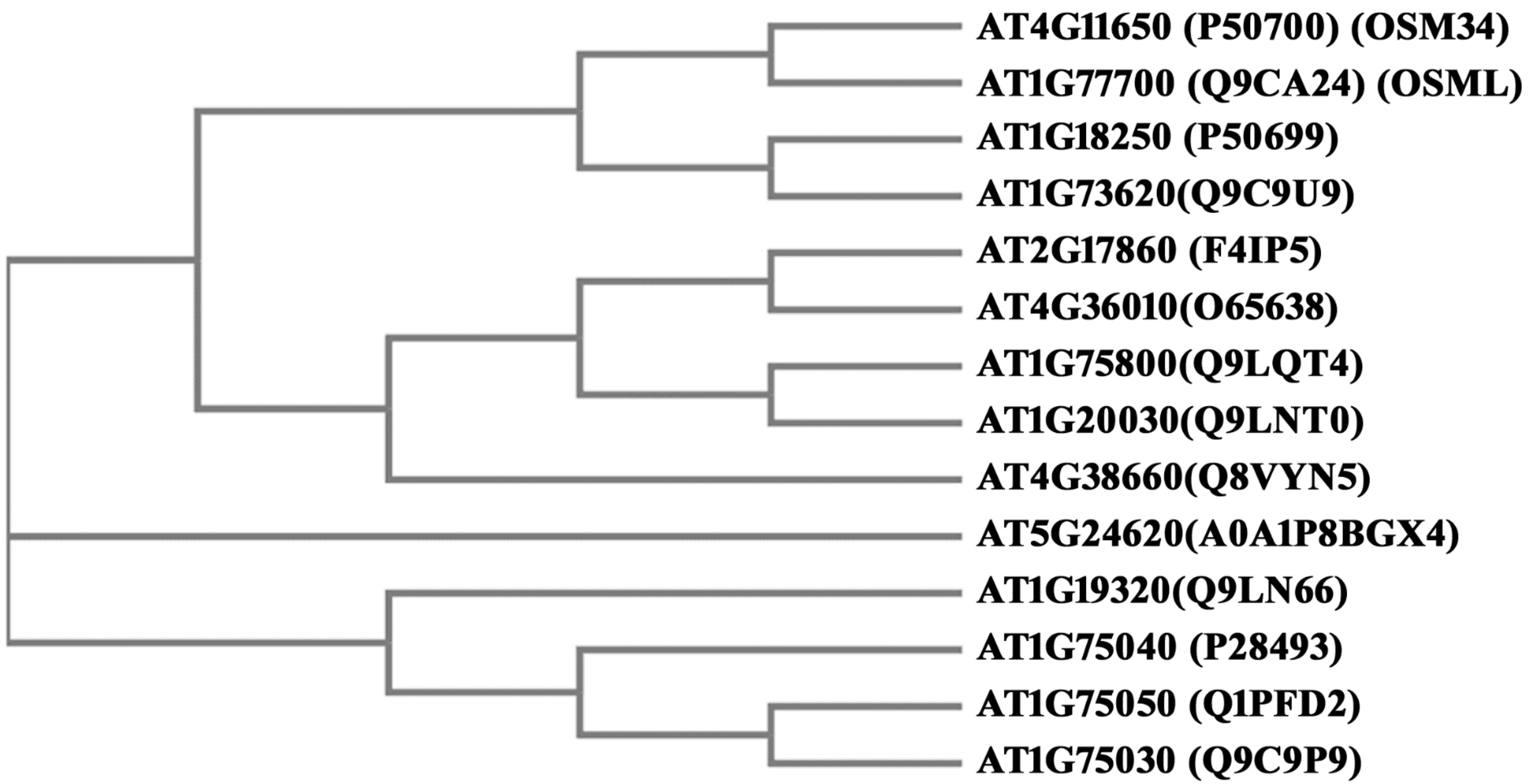

Figure S1. Phylogenetic tree of OSM34 and similar proteins Protein sequences were aligned using Clustal Omega [1]. Each protein sequence was obtained from the Uniprot database. AGI (Protein ID)

1) Madeira, F.; Park, Y.M.; Lee, J.; Buso, N.; Gur, T.; Madhusoodanan, N.; Basutkar, P.; Tivey, A.R.N.; Potter, S.C.; Finn, R.D.; *et al*. The EMBL-EBI search and sequence analysis tools APIs in 2019. *Nucleic Acids Res,* **2019**, 47, W636-W641, doi:10.1093/nar/gkz268.

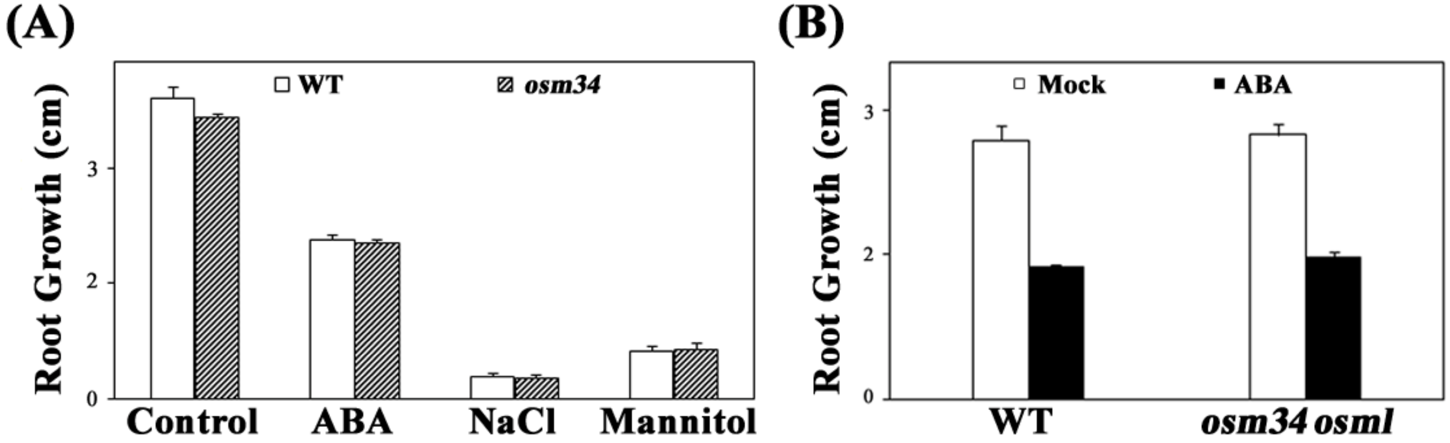

Figure S2. Root growth inhibition caused by abiotic stresses was not affected by the mutation in *OSM34* (A) Five-day-old seedlings of wildtype (Col-0) and *osm34* were transferred to each media containing 30 μM ABA, 150 mM NaCl, and 300 mM Mannitol, and grown for 3 days. **(B)** Five-day-old seedlings of wildtype (Col-0) and *osm34 osml* were transferred to media containing 30 μM ABA and grown for 3 days.

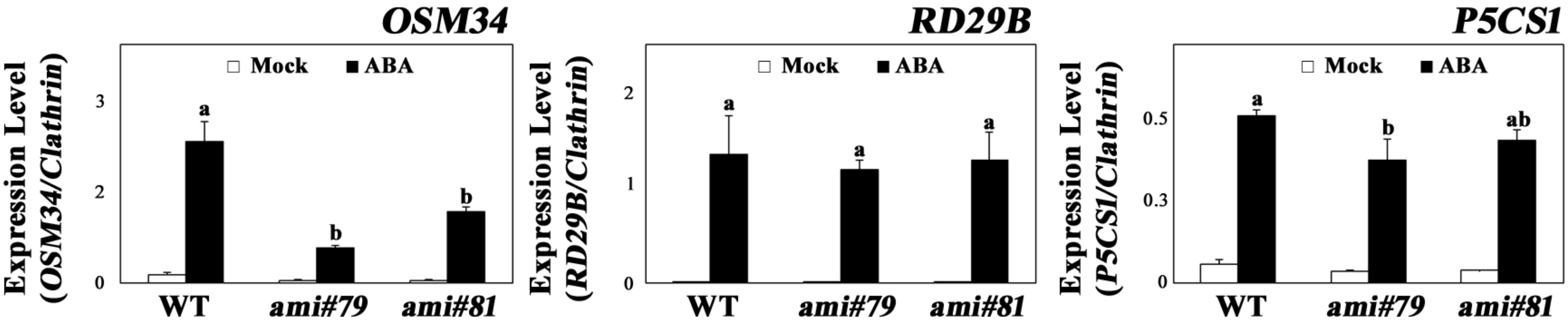

Figure S3. The *OSM34* knock-down lines generated by an *amiRNA* approach shows reduced ABA-induction of gene expression

Homozygous T3 generations of *amiOSM34* #79/81 were used for quantitative PCR analyses. Six-day-old seedlings were treated with 50 μM ABA for 24h. a/ab/b indicate different groups based on the one-way ANOVA Duncan post-hoc analyses ($p < 0.05$).

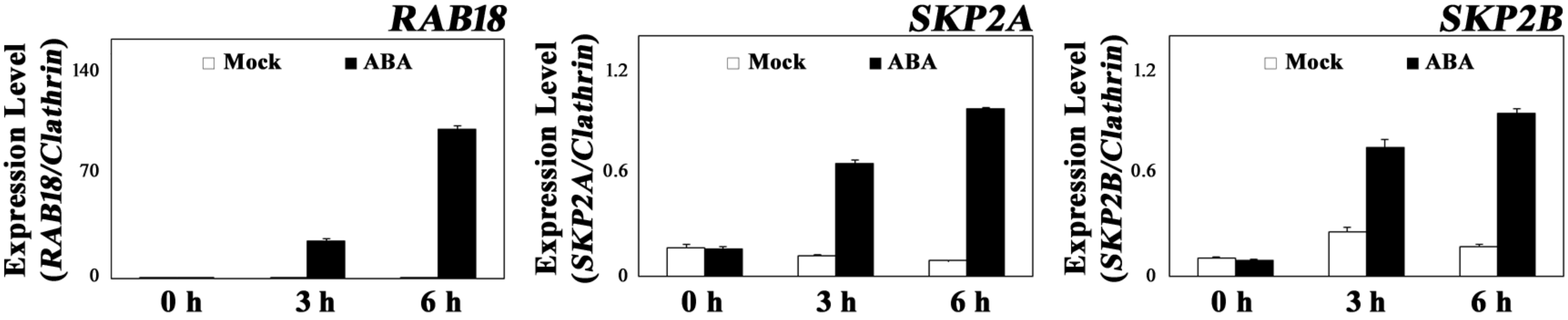

Figure S4. F-Box genes *SKP2A* and *SKP2B* are transcriptionally induced by ABA treatment

The expressions of ABA-responsive genes *RAB18*, *SKP2A*, and *SKP2B* were quantified using quantitative PCR analyses. Eight-day-old seedlings of wildtype (Col-0) were treated with 50 μM ABA for the indicated time (0/3/6 hours).
